# Magnetic Intramedullary Lengthening Nails for Complex Lower Extremity Reconstruction: Functional Outcomes, Bone Healing, and Complications

**DOI:** 10.3390/jcm15166265

**Published:** 2026-08-13

**Authors:** Muhammed Ergün, Ferhat Güler

**Affiliations:** 1 Department of Orthopaedics and Traumatology, Antalya Training and Research Hospital, 07100 Antalya, Türkiye; 2 Department of Orthopaedics and Traumatology, Private Anatolia Hospital, 07100 Antalya, Türkiye; drferhatguler@gmail.com

**Keywords:** magnetic intramedullary lengthening nail, distraction osteogenesis, limb reconstruction, deformity correction, PRECICE system, lower extremity deformity

## Abstract

**Background:** Magnetically controlled intramedullary lengthening nails have become increasingly popular for lower extremity reconstruction because they reduce complications associated with external fixation and facilitate earlier rehabilitation. However, comprehensive data integrating deformity correction, bone healing, functional recovery, psychosocial outcomes, and complication-related analyses remain limited. This study aimed to evaluate the radiological, functional, psychosocial, and complication-related outcomes of magnetic intramedullary lengthening nails in patients undergoing complex lower extremity reconstruction. **Methods:** This retrospective case series included 34 patients who underwent 37 magnetic intramedullary lengthening procedures between 2015 and 2024. Radiological outcomes, bone healing indices, lower extremity alignment parameters, complications, and patient-reported outcomes were evaluated. Functional and psychosocial assessments included the Lower Extremity Functional Scale (LEFS), Short Form-36 (SF-36), and Rosenberg Self-Esteem Scale. Subgroup and correlation analyses were performed to investigate factors associated with regenerate maturation and postoperative complications. **Results:** The mean follow-up duration was 44.6 ± 19.8 months. Radiological union was achieved in all patients. The mean achieved lengthening was 4.8 ± 1.9 cm, while the mean consolidation and distraction indices were 57.6 ± 44.5 days/cm and 1.0 ± 0.02 mm/day, respectively. Tibial lengthening procedures demonstrated significantly higher maturation indices compared with femoral procedures (60.5 ± 37.3 vs. 45.0 ± 45.4 days/cm, *p* = 0.045). Complications occurred in 18.9% of procedures and were associated with significantly prolonged consolidation and maturation indices. Delayed union was the most common complication and was successfully managed with autologous bone grafting or conversion to a standard intramedullary nail when necessary. No implant-related mechanical failure, permanent neurological deficit, or failure of final bone union was observed. Increasing age demonstrated significant positive correlations with both consolidation and maturation indices. Functional and psychosocial outcomes were favorable, with a mean LEFS score of 81.5 ± 15.5% and satisfactory SF-36 and Rosenberg Self-Esteem scores at final follow-up. **Conclusions:** Magnetically controlled intramedullary lengthening nails provide reliable deformity correction, successful bone healing, and satisfactory functional and psychosocial outcomes in patients undergoing complex lower extremity reconstruction. Although complications may prolong regenerate maturation, favorable clinical and radiological outcomes can be achieved with appropriate patient selection, standardized rehabilitation, and timely secondary interventions.

## 1. Introduction

Lower extremity deformities and limb length discrepancies are complex orthopedic conditions associated with impaired gait mechanics, functional limitation, pain, and reduced quality of life. In severe or long-standing cases, these deformities may result in progressive musculoskeletal dysfunction, cosmetic dissatisfaction, and psychosocial distress, substantially affecting daily activities and social participation [1,2,3,4].

Distraction osteogenesis has become a cornerstone technique in limb lengthening and deformity reconstruction. Conventional circular and monolateral external fixation systems provide reliable deformity correction and satisfactory union rates [5,6]. However, prolonged external fixation is associated with considerable morbidity, including pin tract infection, soft tissue irritation, joint stiffness, pain, muscle tethering, and reduced patient tolerance [7,8]. In addition, the psychosocial burden and prolonged rehabilitation associated with external fixation may negatively affect patient satisfaction and overall quality of life.

In recent years, fully implantable limb lengthening systems have gained increasing popularity because of their ability to minimize complications associated with external fixation and facilitate early rehabilitation [9]. Advances in implant technology have led to the widespread use of motorized magnetic intramedullary lengthening nails, particularly the PRECICE system, which enables controlled and noninvasive distraction through an externally regulated magnetic mechanism [7,10,11]. Previous studies evaluating magnetic intramedullary lengthening nails have generally reported favorable radiographic outcomes, reliable regenerate formation, and relatively low complication rates [12,13,14,15,16,17].

Nevertheless, successful lower extremity reconstruction cannot be defined solely by radiographic correction or bone healing. Restoration of physical function, improvement in quality of life, psychosocial recovery, and complication prevention are equally important determinants of treatment success from the patient perspective [18,19]. Although previous studies evaluating magnetic intramedullary lengthening nails have generally focused on radiographic outcomes and limb length restoration, data integrating simultaneous deformity correction, regenerate maturation characteristics, complication-related healing analyses, and patient-reported psychosocial outcomes remain limited in the current literature. Furthermore, comparative analyses between femoral and tibial lengthening procedures and investigations of factors associated with delayed consolidation and complication development are still insufficiently explored. Given the increasing use of magnetic intramedullary lengthening systems in complex lower extremity reconstruction, a more comprehensive evaluation of both radiological and patient-centered outcomes is required.

Therefore, the aim of the present study was to evaluate the radiological outcomes, bone healing characteristics, functional recovery, psychosocial outcomes, and complications associated with magnetic intramedullary lengthening nails in patients undergoing complex lower extremity reconstruction. In addition to deformity correction and limb length restoration, patient-reported functional outcomes and psychosocial recovery were assessed using validated clinical outcome measures, including the Lower Extremity Functional Scale (LEFS), Short Form-36 (SF-36), and Rosenberg Self-Esteem Scale [20,21,22]. Furthermore, subgroup and correlation analyses were performed to investigate factors associated with bone healing, regenerate maturation, and postoperative complications.

## 2. Materials and Methods

### 2.1. Study Design and Patient Selection

This retrospective case series included patients who underwent magnetic intramedullary lengthening nail surgery for lower extremity deformity and/or limb length discrepancy at the Department of Orthopaedics and Traumatology, Antalya Training and Research Hospital, between 2015 and 2024. Inclusion criteria were femoral and/or tibial deformity or shortening associated with limping, pain, or gait disturbance; age ≥10 years; adequate bone morphology for intramedullary lengthening; ambulatory status; and regular postoperative follow-up. Many patients presented with combined angular deformity, mechanical axis deviation, and limb length discrepancy requiring comprehensive deformity reconstruction rather than isolated lengthening procedures.

The study protocol was approved by the Antalya Training and Research Hospital Ethics Committee (Approval No: 2020/16; 24 March 2020) and conducted in accordance with the Declaration of Helsinki. Written informed consent was obtained from all patients and/or their legal guardians.

A total of 34 patients who underwent 37 lengthening procedures were included, as three patients underwent ipsilateral femoral and tibial lengthening. The minimum follow-up duration was 13 months, with a mean follow-up duration of 44.6 months per procedure.

Patients with active infection, severe muscle weakness, inability to ambulate, advanced adjacent joint stiffness, or joint dislocation precluding surgical treatment were excluded. Preoperative and postoperative radiographic evaluations were performed using a standardized institutional deformity assessment protocol (Supplementary Material Table S1). Radiographic outcomes, bone-healing indices, and complication-related analyses were performed per procedure (*n* = 37), whereas demographic variables and patient-reported outcome measures (LEFS, SF-36, and Rosenberg Self-Esteem Scale) were analyzed per patient (*n* = 34). Because three patients underwent both femoral and tibial lengthening procedures, patient-reported outcomes were not included in procedure-level subgroup analyses to avoid non-independence of observations.

### 2.2. Preoperative Evaluation and Deformity Analysis

Preoperative evaluation was performed using standing long-leg orthoroentgenograms and standard anteroposterior/lateral radiographs of the lower extremities. Preoperative planning included assessment of limb length discrepancy, deformity characteristics, overall limb alignment, and determination of the osteotomy level using the institutional PACS system (Sectra PACS IDS7 18.2; Sectra AB, Linköping, Sweden).

In skeletally immature patients, predicted remaining growth and planned lengthening amount were calculated using the Multiplier application developed by the Rubin Institute for Advanced Orthopedics [23].

### 2.3. Implant Characteristics

All procedures were performed using the second-generation PRECICE magnetic intramedullary lengthening nail system (NuVasive Specialized Orthopedics Inc., San Diego, CA, USA), a magnetically driven telescopic intramedullary nail allowing controlled noninvasive distraction [24].

Implant diameter was selected based on preoperative radiographic measurement of the intramedullary canal. During surgery, the canal was reamed up to 2 mm larger than the planned nail diameter, and a nail 2 mm smaller than the final reamed diameter was inserted according to standard intramedullary lengthening principles.

### 2.4. Surgical Technique

All procedures were performed by a single surgeon with more than 12 years of experience in deformity reconstruction and limb lengthening surgery, including approximately 40 magnetic intramedullary lengthening nail procedures and more than 80 limb lengthening procedures using both internal and external fixation techniques prior to and during the study period. Femoral lengthening was carried out using antegrade or retrograde techniques according to deformity location and patient anatomy, whereas tibial lengthening was performed through an antegrade tibial approach. For femoral procedures, antegrade nails were preferred when the osteotomy site was located in the proximal or mid-diaphyseal femur, whereas retrograde nails were used when the deformity correction or osteotomy was located closer to the distal femur. All tibial lengthening procedures were performed using an antegrade tibial nail. Following preoperative planning, osteotomy and intramedullary canal preparation were performed under fluoroscopic guidance, and the PRECICE magnetic intramedullary lengthening nail was inserted using standard surgical principles. Temporary external fixation and/or blocking screws were used when necessary to maintain alignment, rotational stability, and accurate deformity correction during nail insertion [25,26].

### 2.5. Postoperative Protocol

A latency period of 7 days was applied before distraction, followed by distraction at a rate of 1 mm/day in four increments of 0.25 mm. Patients underwent clinical and radiographic follow-up at 2-week intervals during the distraction phase. Weight-bearing progression was determined according to regenerate quality and nail diameter, with full weight-bearing permitted after radiographic cortical consolidation. Functional rehabilitation and range-of-motion exercises were initiated on the first postoperative day.

No weight-bearing was permitted during the distraction phase. Partial weight-bearing was gradually initiated during the consolidation phase according to regenerate quality and implant diameter. Patients treated with 8.5 mm and 10.7 mm nails were instructed not to exceed approximately one-third of their body weight during partial weight-bearing, whereas those treated with 12.5 mm nails were allowed up to approximately one-half of their body weight. Weight-bearing was progressively increased from toe-touch loading to the prescribed limits according to radiographic and clinical progression. Full weight-bearing was permitted after radiographic evidence of consolidation in at least three cortices.

### 2.6. Outcome Assessment

Radiographic and functional outcomes were evaluated after bone healing and at final follow-up. Standing long-leg orthoroentgenograms and standard anteroposterior/lateral radiographs of the lower extremities were obtained in all patients. Radiographic assessment focused on regenerate formation, bone healing, restoration of limb length, and coronal plane alignment using the institutional PACS system (Sectra PACS IDS7 18.2; Sectra AB, Sweden).

Mechanical lateral proximal femoral angle (mLPFA), mechanical lateral distal femoral angle (mLDFA), mechanical medial proximal tibial angle (mMPTA), and mechanical lateral distal tibial angle (mLTDA) were measured preoperatively and postoperatively to evaluate lower extremity alignment correction.

Bone healing was assessed using distraction, maturation, and consolidation indices [9]. The distraction index was defined as the amount of lengthening achieved per day (mm/day). The maturation index was calculated as the time from the end of distraction to radiographic cortical maturation, defined as bridging callus formation in at least three cortices on anteroposterior and lateral radiographs, per centimeter of lengthening. The consolidation index was defined as the time from osteotomy to radiographic consolidation, characterized by cortical continuity and sufficient regenerate mineralization permitting full weight-bearing, per centimeter of lengthening.

Complications and secondary interventions were recorded throughout follow-up and included delayed union, fracture, neurological complications, implant-related complications, and secondary surgical interventions. Complications were additionally classified according to the Paley classification system as problems, obstacles, or true complications based on the need for intervention and persistence after treatment completion [5]. Patient-reported outcome measures were assessed at the final follow-up visit after completion of treatment and radiographic bone healing. Functional outcome was evaluated using the Lower Extremity Functional Scale (LEFS), and psychosocial outcomes were assessed using the Short Form-36 (SF-36) and Rosenberg Self-Esteem Scale. LEFS scores are presented as percentages of the maximum possible score (80 points), with higher scores indicating better functional status. Turkish-language versions of the LEFS, SF-36, and Rosenberg Self-Esteem Scale were used for all assessments.

### 2.7. Statistical Analysis

Statistical analyses were performed using IBM SPSS Statistics for Windows, version 26.0 (IBM Corp., Armonk, NY, USA). Data distribution was assessed using the Shapiro–Wilk test. Continuous variables were presented as mean ± standard deviation or median (minimum–maximum), whereas categorical variables were expressed as frequency and percentage.

Comparisons between independent groups were performed using the Mann–Whitney U test for continuous variables and Fisher’s exact test for categorical variables. Comparisons among more than two groups were conducted using the Kruskal–Wallis test. Preoperative and postoperative radiographic measurements were compared using the Wilcoxon signed-rank test. Correlations between continuous variables were evaluated using Spearman correlation analysis. A *p* value < 0.05 was considered statistically significant.

Given the exploratory nature of the study and the relatively small sample size, no formal adjustment for multiple comparisons was performed. Therefore, subgroup and correlation analyses should be interpreted as exploratory.

## 3. Results

A total of 34 patients who underwent 37 magnetic intramedullary lengthening procedures were included in the study. The mean age of the cohort was 24.1 ± 10.3 years (range, 12–51 years), and 24 patients (70.6%) were male. Baseline demographic and clinical characteristics are summarized in Table 1.

The right lower extremity was involved in 22 procedures (59.5%), whereas the left lower extremity was involved in 15 procedures (40.5%). Femoral lengthening was performed in 30 procedures (81.1%) and tibial lengthening in 7 procedures (18.9%). Post-traumatic deformities represented the most common etiology (56.8%), followed by congenital deformities (29.7%) and poliomyelitis sequelae (13.5%). Regarding the surgical technique, femoral antegrade nail (FAN), femoral retrograde nail (FRN), and tibial nail (TN) were used in 13 (35.1%), 17 (45.9%), and 7 procedures (18.9%), respectively. The mean follow-up duration was 44.6 ± 19.8 months.

Radiological and functional outcomes are summarized in Table 2. Radiological union was achieved in all patients. The mean achieved lengthening was 4.8 ± 1.9 cm. The mean consolidation and distraction indices were 57.6 ± 44.5 days/cm and 1.0 ± 0.02 mm/day, respectively. Maturation characteristics were further evaluated in subgroup and complication-related analyses.

Functional outcome assessment demonstrated satisfactory postoperative recovery. The mean LEFS score was 81.5 ± 15.5%, whereas the mean Rosenberg Self-Esteem score was 34.2 ± 4.4. SF-36 subscale scores also demonstrated favorable physical, emotional, and social health outcomes at final follow-up, particularly in pain, social functioning, and general health domains (Table 2).

Continuous variables are presented as mean ± standard deviation. LEFS: Lower Extremity Functional Scale; SF-36: 36-Item Short Form Health Survey.Radiographic alignment analysis demonstrated postoperative changes in coronal plane parameters following deformity correction (Table 3). A statistically significant change was observed in mLPFA measurements (*p* < 0.001), whereas changes in mLDFA, mMPTA, and mLTDA did not reach statistical significance. Postoperative mLDFA, mMPTA, and mLTDA values were close to contralateral limb values, suggesting maintenance or restoration of coronal plane alignment. Restoration of limb length was achieved in all patients. At final follow-up, the median residual limb length discrepancy was 0 mm, and the mean residual discrepancy was −1.4 ± 5.7 mm.

Comparisons between femoral and tibial lengthening procedures are summarized in Table 4. Patients undergoing tibial lengthening demonstrated higher consolidation and maturation indices compared with those undergoing femoral lengthening. Although the consolidation index was higher in the tibial group, the difference did not reach statistical significance (71.8 ± 36.6 vs. 54.3 ± 45.9 days/cm, *p* = 0.121). In contrast, the maturation index was significantly higher in tibial procedures compared with femoral procedures (60.5 ± 37.3 vs. 45.0 ± 45.4 days/cm, *p* = 0.045).

Complication-related comparisons are summarized in Table 5. Complications were observed in 7 procedures (18.9%). Procedures classified as having complications showed higher consolidation and maturation indices than those without complications; however, this finding should be interpreted cautiously because delayed union, the most frequent complication, is inherently associated with prolonged healing indices. The mean consolidation index was significantly higher in the complication group than in the non-complication group (89.9 ± 51.2 vs. 50.0 ± 39.9 days/cm, *p* = 0.021). Similarly, the maturation index was significantly higher among patients with complications (79.4 ± 51.2 vs. 40.6 ± 39.5 days/cm, *p* = 0.017). Complications included delayed union in three patients, all of whom underwent femoral lengthening procedures. Two patients were successfully treated with autologous bone grafting, whereas one required conversion to a standard intramedullary nail. One 12-year-old male patient without comorbidities who underwent antegrade femoral lengthening developed postoperative infection characterized by serohemorrhagic wound drainage. Microbiological cultures were not obtained because the drainage resolved rapidly without systemic signs of infection. The patient was treated with intravenous piperacillin–tazobactam for 7 days, resulting in complete resolution of symptoms without additional surgical intervention. No recurrence was observed during follow-up. According to the Paley classification, transient neurological deficits were considered problems because they resolved without surgical intervention, whereas delayed union requiring bone grafting or nail exchange and infection requiring treatment were classified as obstacles (Table 6). No true complication resulting in permanent sequelae was observed. No significant differences were observed between groups regarding age, sex distribution, smoking history, operated bone segment, or comorbidity status.

Correlation analysis is summarized in Table 7. Spearman correlation analysis demonstrated a moderate positive correlation between age and consolidation index (r = 0.446, *p* = 0.006), indicating prolonged bone healing duration with increasing age. Similarly, age demonstrated a significant positive correlation with maturation index (r = 0.366, *p* = 0.026). In contrast, no significant correlation was identified between LEFS and Rosenberg Self-Esteem scores (r = 0.239, *p* = 0.154).

Values are presented as mean ± standard deviation unless otherwise indicated. Mann–Whitney U test was used for two-group comparisons, Kruskal–Wallis test for multiple-group comparisons, Fisher’s exact test for categorical variables, and Spearman correlation analysis for continuous variables. A *p* value of <0.05 was considered statistically significant.

**Figure 1:**
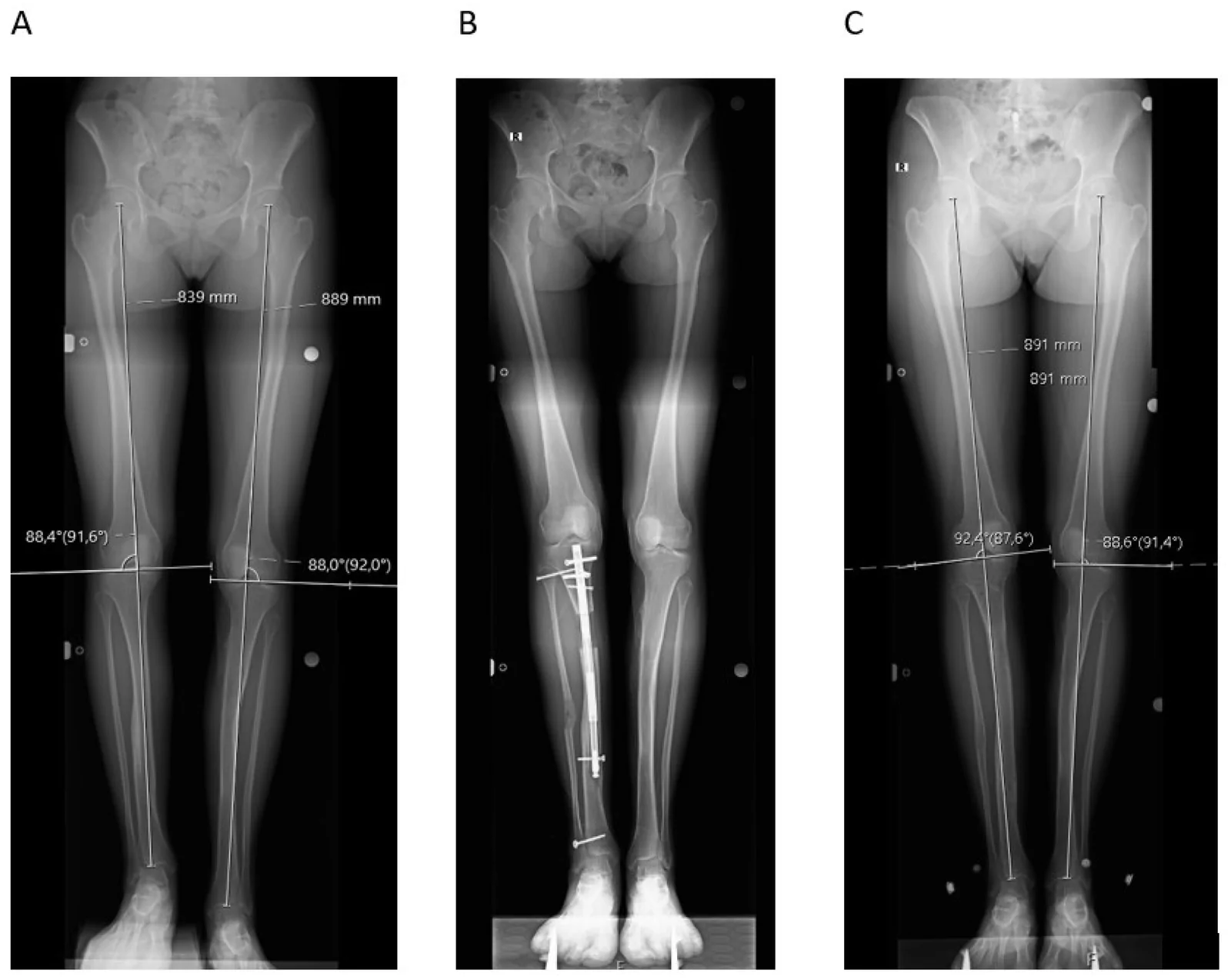
Representative case of tibial lengthening and deformity correction using a magnetically controlled intramedullary nail in a 17-year-old patient with lower extremity length discrepancy. (**A**) Preoperative standing long-leg radiograph demonstrating tibial shortening, limb length discrepancy, and coronal plane deformity. Preoperative alignment parameters included an mLDFA of 92.4° and an mMPTA of 88.6°. (**B**) Radiograph obtained during the distraction/consolidation phase following tibial osteotomy and insertion of a magnetically controlled intramedullary lengthening nail. (**C**) Final standing long-leg radiograph after completion of treatment demonstrating successful limb length equalization, satisfactory regenerate consolidation, restoration of lower extremity alignment, and improvement of the coronal plane deformity. Final alignment parameters were mLDFA 88.4° and mMPTA 88.0°.

A representative case demonstrating simultaneous deformity correction and tibial lengthening using a magnetically controlled intramedullary nail is presented in Figure 1.

**Figure 2:**
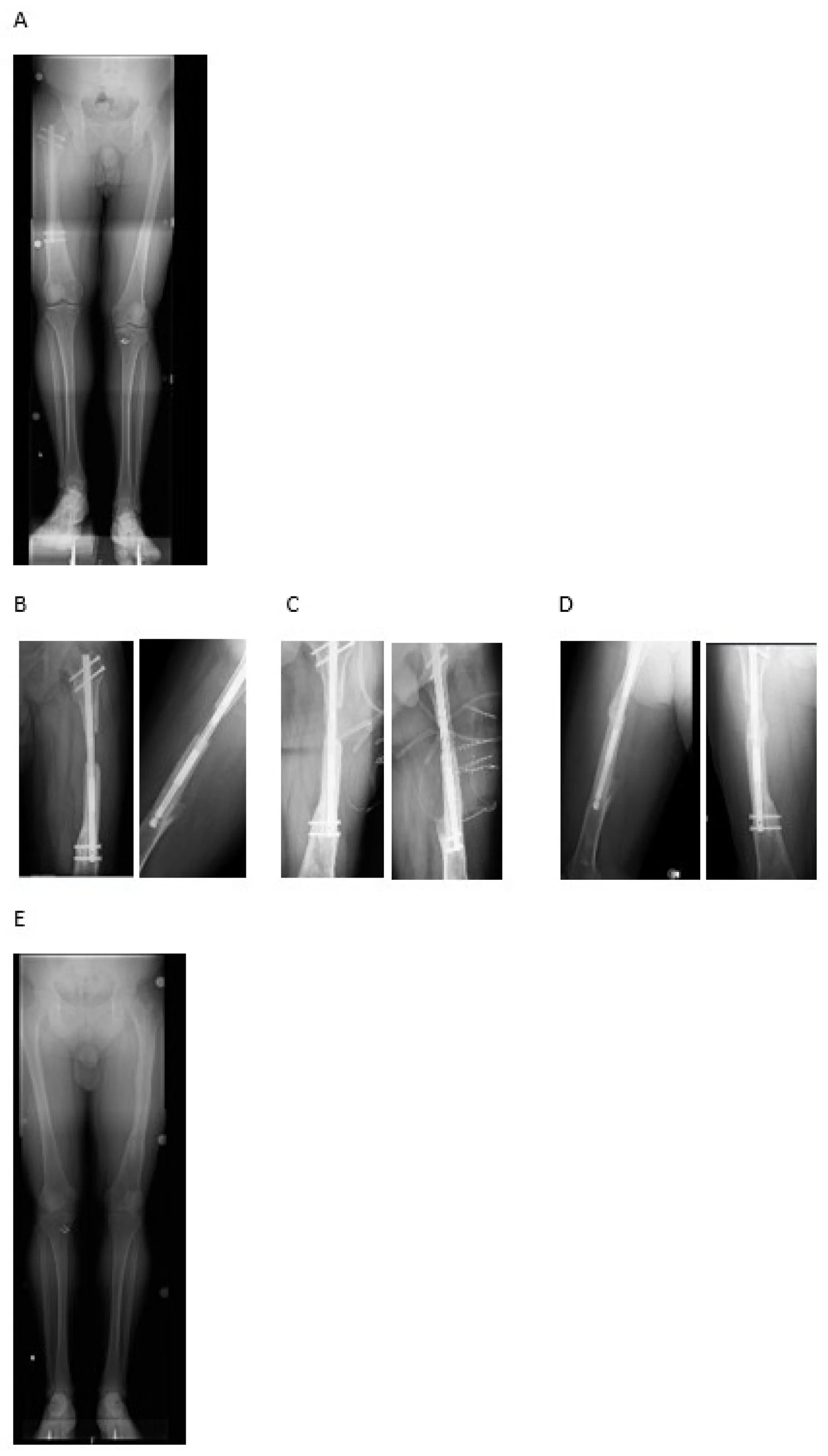
Representative case of delayed union following femoral lengthening requiring. autologous bone grafting and demonstrating subsequent successful union. (**A**) Immediate postoperative anteroposterior radiograph following tibial osteotomy and implantation of a magnetically controlled intramedullary lengthening nail. (**B**) Anteroposterior and lateral radiographs obtained 7 months postoperatively demonstrating delayed union with insufficient regenerate consolidation at the osteotomy site. (**C**) Anteroposterior and lateral radiographs obtained 8 months postoperatively following autologous bone grafting performed at the delayed union site to stimulate bone healing. (**D**) Anteroposterior and lateral radiographs at 1-year follow-up demonstrating progressive regenerate maturation and bridging callus formation after bone grafting. (**E**) Final standing long-leg radiograph obtained 3 years postoperatively demonstrating complete union, satisfactory regenerate consolidation, maintenance of alignment, and successful limb length restoration.

A representative case of delayed union requiring autologous bone grafting and demonstrating subsequent successful union is presented in Figure 2.

## 4. Discussion

Magnetically controlled intramedullary lengthening nails provided reliable deformity correction, successful bone healing, and satisfactory functional recovery in patients undergoing complex lower extremity reconstruction in the present study. Radiological union was achieved in all patients, and postoperative functional and psychosocial outcome scores demonstrated favorable mid-term recovery. Although complications occurred in a subset of patients, all complications were successfully managed without permanent sequelae or failure of bone healing. In addition, tibial lengthening procedures showed higher maturation indices than femoral procedures. However, this finding should be interpreted cautiously because only seven tibial procedures were included, limiting the statistical power of this subgroup comparison. Furthermore, increasing age was associated with delayed bone healing parameters. The observed correlation between increasing age and prolonged consolidation and maturation indices may support the influence of patient-related biological factors on regenerate healing capacity. The significant postoperative change observed in mLPFA most likely reflects correction of proximal femoral deformities present in a subset of patients rather than systematic overcorrection. Given the heterogeneous deformity patterns included in the study, changes in mLPFA should be interpreted in the context of individualized deformity correction strategies.

Although motorized intramedullary nails have substantially improved patient comfort and reduced many complications traditionally associated with external fixation, device-related mechanical failures remain an important concern in limb lengthening surgery. Fully internal limb lengthening techniques have gained increasing popularity because they facilitate earlier rehabilitation and reduce complications associated with prolonged external fixation, including pin-site infections and soft-tissue tethering [9]. Nevertheless, previous studies have reported complications such as motor dysfunction, backtracking, and failure of the telescopic mechanism, some of which required unplanned revision procedures and negatively affected treatment outcomes [27,28,29]. In the present study, however, no implant-related mechanical complication was observed throughout follow-up. This finding may reflect careful patient selection, meticulous surgical technique, and appropriate adherence to distraction protocols. Nevertheless, given the relatively limited sample size and heterogeneity among available implant systems, larger studies are still required to better define the true incidence and predictors of device-related mechanical failure.

Despite the clinical advantages of magnetically controlled intramedullary lengthening nails, disparities in healthcare access and economic resources continue to limit their widespread availability, particularly in developing countries. In Turkey, the use of these systems remains relatively limited because of restricted reimbursement policies, narrow clinical indications, and the high economic burden associated with these implants. Consequently, access to fully internal lengthening technologies may remain challenging despite their potential functional and psychosocial benefits. These limitations may partially explain the relatively low number of patients treated with fully internal lengthening systems in many centers.

Despite advances in magnetically controlled intramedullary lengthening systems, complication management remains a critical aspect of lower extremity reconstruction surgery. In the present study, complications were observed in 18.9% of procedures, and patients who developed complications demonstrated significantly prolonged consolidation and maturation indices compared with those without complications. Delayed union represented the most frequent complication and was observed in three patients, two of whom were successfully treated with autologous bone grafting, whereas one required conversion to a standard intramedullary nail. In addition, one patient developed infection during follow-up, and transient neurological deficits were observed in three patients. Importantly, no implant-related mechanical failure, permanent neurological deficit, or failure of final bone union was encountered. Similarly, Talmaç et al. reported that complications requiring reoperation were relatively infrequent and generally manageable in patients treated with magnetic intramedullary lengthening nails [30]. In their series, reoperations were required for mechanical axis correction, distraction mechanism failure, and ankle equinus contracture; however, all cases were successfully resolved without long-term functional impairment. Previous studies have reported higher complication and reoperation rates, particularly in cohorts involving complex congenital deformities and multiplanar reconstructions [13,14]. In contrast, the relatively favorable complication profile observed in our series may be associated with careful patient selection, standardized rehabilitation protocols, accumulated surgical experience, and the avoidance of external fixation-related complications such as pin-site infections and soft-tissue tethering. Collectively, these findings suggest that although complications may prolong regenerate maturation and consolidation, satisfactory clinical and radiological outcomes can still be achieved with timely recognition, appropriate secondary interventions, and close postoperative follow-up. The association between complication status and prolonged consolidation or maturation indices should be interpreted with caution, because delayed union was included as a complication and is intrinsically related to delayed radiographic healing. Therefore, this analysis should be considered descriptive rather than evidence of an independent predictive relationship between complications and delayed regenerate maturation.

Despite the complexity of the deformities treated in our cohort, the overall complication profile remained acceptable, and all patients ultimately achieved radiological union. More than half of the procedures were performed for post-traumatic deformities, while nearly one-third involved congenital etiologies, both of which may present substantial biological and technical challenges during deformity correction and limb reconstruction. Nevertheless, satisfactory functional recovery was achieved, with a mean LEFS score of 81.5% and favorable SF-36 and Rosenberg Self-Esteem scores at final follow-up. Although complications were associated with significantly prolonged consolidation and maturation indices, no permanent neurological deficit, implant-related mechanical failure, or failure of final bone healing was encountered. Previous studies have suggested that congenital deformities and multiplanar reconstructions may be associated with higher complication rates and delayed regenerate maturation compared with acquired deformities [15,31]. However, no clear pattern of impaired consolidation was observed among congenital cases in our series despite the heterogeneity of etiologies. This finding suggests that careful preoperative planning, individualized deformity analysis, and standardized postoperative rehabilitation protocols may help achieve reliable regenerate formation and satisfactory functional recovery even in anatomically challenging deformities.

The present study has several strengths. First, the mean follow-up duration was relatively long, allowing comprehensive evaluation of both radiological union and mid-term functional outcomes. Second, unlike many previous series focusing primarily on isolated limb length discrepancy, our cohort included patients undergoing simultaneous deformity correction and limb lengthening, reflecting the complexity of real-world lower extremity reconstruction practice. Third, multiple validated functional and psychosocial outcome measures, including LEFS, Rosenberg Self-Esteem Scale, and SF-36 subscales, were evaluated comprehensively. In addition, all procedures were performed by the same experienced surgical team using standardized surgical and rehabilitation protocols, which may have reduced treatment variability. Nevertheless, several limitations should be acknowledged. The retrospective study design and relatively limited sample size may restrict the generalizability of the findings and limit subgroup analyses according to deformity etiology or surgical technique. Furthermore, the heterogeneity of deformity etiologies and reconstruction procedures may have influenced radiological and functional outcomes. In addition, the relatively small number of tibial lengthening procedures may have limited the statistical power of subgroup comparisons between femoral and tibial reconstruction procedures. Despite these limitations, the present findings suggest that magnetically controlled intramedullary lengthening systems can provide reliable deformity correction, successful bone healing, and satisfactory functional and psychosocial recovery in carefully selected patients undergoing complex lower extremity reconstruction.

## 5. Conclusions

Magnetically controlled intramedullary lengthening systems provided reliable deformity correction, successful bone healing, and satisfactory functional and psychosocial outcomes in patients undergoing complex lower extremity reconstruction. Despite the occurrence of complications in a subset of patients, all complications were successfully managed without permanent sequelae, and radiological union was achieved in all cases. Tibial lengthening procedures tended to demonstrate higher maturation indices than femoral procedures; however, this observation should be interpreted cautiously given the limited number of tibial procedures included in the study. In addition to effective limb length restoration, these systems enabled simultaneous correction of associated deformities while maintaining favorable mid-term clinical outcomes. Although larger prospective studies are required to further clarify factors influencing regenerate maturation and complication risk, the present findings support the safe and effective use of magnetically controlled intramedullary lengthening nails in appropriately selected patients requiring complex lower extremity reconstruction.

## Figures and Tables

**Table 1:** Demographic and Clinical Characteristics of the Patients.

Variable	Value
Number of patients/procedures	34/37
Age, years	24.1 ± 10.3
Male/Female	24/10
Height, cm	171.7 ± 12.4
Weight, kg	71.8 ± 18.7
Smoking history, *n* (%)	11 (32.4)
Comorbidity, *n* (%) **	3 (8.8)
Right/Left side	22/15
Femur/Tibia	30/7
Etiology, *n* (%)	
• Post-traumatic	21 (56.8)
• Congenital	11 (29.7)
• Poliomyelitis sequelae	5 (13.5)
FAN/FRN/TN	13/17/7
Follow-up duration, months	44.6 ± 19.8

Values are presented as mean ± standard deviation or number of patients (%). FAN: Femur antegrade nail; FRN: Femur retrograde nail; TN: Tibial nail. ** Comorbidities consisted of diabetes mellitus and hypertension in two patients and asthma in one patient.

**Table 2:** Radiological and Functional Outcomes.

Variable	Value
Union rate	100%
Consolidation index, days/cm	57.6 ± 44.5
Distraction index, mm/day	1.0 ± 0.02
Achieved lengthening, cm	4.8 ± 1.9
LEFS, %	81.5 ± 15.5
Rosenberg score	34.2 ± 4.4
SF-36 physical function	75.4 ± 20.1
SF-36 physical role difficulty	79.7 ± 32.7
SF-36 emotional role difficulty	78.4 ± 34.4
SF-36 energy	76.8 ± 18.4
SF-36 mental health	76.6 ± 16.7
SF-36 social functioning	84.8 ± 24.3
SF-36 pain	89.1 ± 17.4
SF-36 general health	80.0 ± 16.5

**Table 3:** Radiographic Alignment Parameters.

Variable	Contralateral Limb	Preoperative Pathologic Limb	Postoperative Pathologic Limb	*p* Value *
mLPFA, °	90.1 ± 10.7	89.6 ± 18.3	97.0 ± 12.7	<0.001
mLDFA, °	87.7 ± 3.3	88.2 ± 7.1	88.5 ± 4.5	0.114
mMPTA, °	87.9 ± 2.1	89.5 ± 5.7	88.1 ± 3.6	0.198
mLTDA, °	86.9 ± 4.7	85.7 ± 7.8	86.9 ± 4.2	0.465

* Values are presented as mean ± standard deviation. *p* values represent comparisons between preoperative and postoperative pathologic limb measurements. mLPFA: Mechanical lateral proximal femoral angle; mLDFA: Mechanical lateral distal femoral angle; mMPTA: Mechanical medial proximal tibial angle; mLTDA: Mechanical lateral distal tibial angle, °: degrees.

**Table 4:** Comparison of Femoral and Tibial Lengthening Procedures.

Variable	Femur (*n* = 30)	Tibia (*n* = 7)	*p* Value
Consolidation index, days/cm	54.3 ± 45.9	71.8 ± 36.6	0.121
Maturation index, days/cm	45.0 ± 45.4	60.5 ± 37.3	0.045

Values are presented as mean ± standard deviation. Comparisons were performed using the Mann–Whitney U test.

**Table 5:** Comparison According to Complication Status.

Variable	Complication (+) (*n* = 7)	Complication (−) (*n* = 30)	*p* Value
Age, years	23.7 ± 8.1	24.1 ± 10.9	0.800
Male/Female	6/1	21/9	0.647
Smoking history, *n* (%)	1 (14.3)	10 (33.3)	0.649
Femur/Tibia	5/2	25/5	0.596
Comorbidity, *n* (%)	0 (0)	3 (10.0)	1.000
Consolidation index, days/cm	89.9 ± 51.2	50.0 ± 39.9	0.021
Maturation index, days/cm	79.4 ± 51.2	40.6 ± 39.5	0.017

Values are presented as mean ± standard deviation or number (%). Comparisons were performed using the Mann–Whitney U test or Fisher’s exact test, as appropriate.

**Table 6:** Complication Classification According to the Paley System.

Complication	Number of Cases	Paley Classification	Management	Final Outcome
Transient neurological deficit	3	Problem	Conservative follow-up	Resolved completely
Delayed union requiring autologous bone grafting	2	Obstacle	Autologous bone grafting	Union achieved
Delayed union requiring nail exchange	1	Obstacle	Conversion to standard intramedullary nail	Union achieved
Infection	1	Obstacle	Debridement/antibiotic treatment	Resolved
Permanent sequelae	0	True complication	—	None

Complications were classified according to the Paley classification system.

**Table 7:** Correlation Analysis.

Variable Pair	Spearman r	*p* Value
Age—Consolidation index	0.446	0.006
Age—Maturation index	0.366	0.026
LEFS—Rosenberg score	0.239	0.154

Correlation analysis was performed using the Spearman correlation test.

## Data Availability

The data presented in this study are available from the corresponding author upon reasonable request. The data are not publicly available due to privacy and ethical restrictions involving patient information.

## Supplementary Materials

The following supporting information can be downloaded at: https://www.mdpi.com/article/10.3390/jcm15166265/s1, Table S1: Individual Patient Characteristics and Deformity Parameters.

## References

[1] Gurney B. (2002). Leg length discrepancy. Gait Posture.

[2] Gurney B., Mermier C., Robergs R., Gibson A., Rivero D. (2001). Effects of limb-length discrepancy on gait economy and lower-extremity muscle activity in older adults. J. Bone Jt. Surg. Am..

[3] Delacerda F.G., Wikoff O.D. (1982). Effect of lower extremity asymmetry on the kinematics of gait. J. Orthop. Sports Phys. Ther..

[4] Bandi W., Hungerford D.S. (1977). Leg Length Discrepancy; The Injured Knee.

[5] Paley D. (1990). Problems, Obstacles, and Complications of Limb Lengthening by the Ilizarov Technique. Clin. Orthop. Relat. Res..

[6] Prince D.E., Herzenberg J.E., Standard S.C., Paley D. (2015). Lengthening With External Fixation Is Effective in Congenital Femoral Deficiency. Clin. Orthop. Relat. Res..

[7] Tjernström B., Olerud S., Rehnberg L. (1994). Limb lengthening by callus distraction. Complications in 53 cases operated 1980–1991. Acta Orthop. Scand..

[8] Green S.A. (1983). Complications of external skeletal fixation. Clin. Orthop. Relat. Res..

[9] Iobst C.A., Rozbruch S.R., Nelson S., Fragomen A. (2018). Simultaneous acute femoral deformity correction and gradual limb lengthening using a retrograde femoral nail: Technique and clinical results. J. Am. Acad. Orthop. Surg..

[10] Nasto L.A., Coppa V., Riganti S., Ruzzini L., Manfrini M., Campanacci L., Palmacci O., Boero S. (2020). Clinical results and complication rates of lower limb lengthening in paediatric patients using the PRECICE 2 intramedullary magnetic nail. J. Pediatr. Orthop. B.

[11] Schep N.W.L., van Lieshout E.M.M., Patka P., Vogels L.M.M. (2009). Long-term functional and quality of live assessment following post-traumatic distraction osteogenesis of the lower limb. Strateg. Trauma Limb Reconstr..

[12] Black S.R., Kwon M.S., Cherkashin A.M., Samchukov M.L., Birch J.G., Jo C.H. (2015). Length ening in congenital femoral deficiency: A comparison of circular external fixation and a motorized intramedullary nail. J. Bone Jt. Surg. Am..

[13] Frommer A., Rödl R., Gosheger G., Vogt B. (2018). Application of motorized intra medullary lengthening nails in skeletally immature patients: Indications and limitations. Unfallchirurg.

[14] Shabtai L., Specht S.C., Standard S.C., Herzenberg J.E. (2014). Internal lengthening device for congenital femoral deficiency and fibular hemimelia. Clin. Orthop. Relat. Res..

[15] Björn V.O.G.T., Laufer A., Gosheger G., Toporowski G., Antfang C., Roelfing J.D., Robert R.Ö.D.L., Frommer A. (2024). Evaluation of simultaneous bilateral femoral distraction osteogenesis with antegrade intramedullary lengthening nails in achondroplasia with rhizomelic short stature: A retrospective study of 15 patients with a minimum follow-up of 2 years. Acta Orthop..

[16] Laubscher M., Mitchell C., Timms A., Goodier D., Calder P. (2016). Outcomes following femoral lengthening. Bone Jt. J..

[17] Karakoyun O., Sokucu S., Erol M.F., Kucukkaya M., Kabukcuoglu Y.S. (2016). Use of a Magnetic Bone Nail for Lengthening of the Femur and Tibia. J. Orthop. Surg..

[18] Kaastad T.S., Tveter A.T., Steen H., Holm I. (2017). Physical function and health-related quality of life in young adults with unilateral congenital lower-limb deficiencies. J. Child. Orthop..

[19] Kim S.-J., Balce G.C., Agashe M.V., Song S.-H., Song H.-R. (2011). Is Bilateral Lower Limb Lengthening Appropriate for Achondroplasia?: Midterm Analysis of the Complications and Quality of Life. Clin. Orthop. Relat. Res..

[20] Binkley J.M., Stratford P.W., Lott S.A., Riddle D.L. (1999). The Lower Extremity Functional Scale (LEFS): Scale development, measurement properties, and clinical application. Phys. Ther..

[21] McKay M.T., Boduszek D., Harvey S.A. (2014). The Rosenberg Self-Esteem Scale: A bifactor answer to a two-factor question?. J. Pers. Assess..

[22] Ware J.E., Sherbourne C.D. (1992). The MOS 36-item short-form health survey (SF-36). I. Conceptual framework and item selection. Med. Care.

[23] (2019). The Multiplier Application.

[24] Paley D. (2015). PRECICE intramedullary limb lengthening system. Expert Rev. Med. Devices.

[25] Rozbruch S.R., Birch J.G., Dahl M.T., Herzenberg J.E. (2014). Motorized Intramedullary Nail for Management of Limb-length Discrepancy and Deformity. J. Am. Acad. Orthop. Surg..

[26] Krettek C., Miclau T., Schandelmaier P., Stephan C., Mohlmann U., Tscherne H. (1999). The mechanical effect of blocking screws (‘‘Poller screws’’) in stabilizing tibia fractures with short proximal or distal fragments after insertion of small-diameter intramedullary nails. J. Orthop. Trauma.

[27] Giorgino R., Cornacchini J., Sillmann Y.M., Kostyra D., Berkane Y., Peretti G.M., Mangiavini L. (2025). Aesthetic lower limb lengthening techniques: A systematic review of efficacy, complications, and patient satisfaction. J. Orthop. Surg. Res..

[28] Makvana S., Robertson A., Britten S., Calder P. (2024). Consent in Limb Lengthening Surgery: Predicting the True Incidence of Material Risk. Strateg. Trauma Limb Reconstr..

[29] Birch J.G. (2017). A Brief History of Limb Lengthening. J. Pediatr. Orthop..

[30] Talmaç M.A., Civan M., Mendes E. (2025). Clinical and radiological outcomes of magnetically controlled intramedullary lengthening nails in adolescents and young adults: A retrospective cohort study. J. Orthop. Surg. Res..

[31] Al-Sayyad M.J., Alshoaibi F.A., Alshuaibi A.A., Almohammadi A.F., Almaghrabi H.A., Alsaady A.M., Alsayyad J.M., Alshoaibi F., Alimohammadi A.F., Alsaady A. (2025). Results of Lower Limb Bone Lengthening by Using Motorized and Magnet-Driven Intramedullary Nails to Treat Limb Length Discrepancy. Cureus.

