# Supplementary material for: Magnetic Intramedullary Lengthening Nails for Complex Lower Extremity Reconstruction: Functional Outcomes, Bone Healing, and Complications
Source: J Clin Med. 2026 Aug 13;15(16):6265. doi: 10.3390/jcm15166265 (PMC13512922; doi:10.3390/jcm15166265)
Supplement: Supplementary file 1 [file jcm-15-06265-s001.zip › jcm-4371989-supplementary.pdf]

Supplementary Table S1. Individual Patient Characteristics and Deformity Parameters

Patient ID	Etiology	Bone	LLD	mLPFA Pre	mLPFA Post	mLDFA Pre	mLDFA Post	mMPTA Pre	mMPTA Post	mLDTA Pre	mLDTA Post
1	Post-traumatic	Tibia	30	90.0	86.0	87.0	87.5	92.0	90.0	91.0	87.0
2	Post-traumatic	Femur	25	122.0	108.0	90.5	85.0	90.0	85.0	88.0	87.5
3	Poliomyelitis sequelae	Femur	55	62.0	75.0	91.0	91.0	92.0	91.4	90.0	82.5
4	Post-traumatic	Femur	90	94.0	91.0	105.5	92.0	105.0	91.0	96.5	90.5
5	Post-traumatic	Femur	40	99.5	114.0	90.5	91.0	91.5	89.5	92.5	88.0
6	Poliomyelitis sequelae	Femur	85	92.5	92.0	88.0	92.0	87.5	92.0	63.5	78.5
7	Poliomyelitis sequelae	Tibia	85	92.5	92.0	88.0	92.0	87.5	92.0	63.5	78.5
7	Post-traumatic	Femur	60	92.5	102.0	90.0	88.0	87.0	86.0	88.0	85.0
9	Post-traumatic	Tibia	45	86.0	85.0	88.5	91.5	87.5	91.0	84.0	85.5
10	Congenital	Femur	110	86.8	96.7	80.1	86.1	91.2	88.8	88.1	89.9
11	Congenital	Tibia	110	86.8	96.7	80.1	86.1	91.2	88.8	88.1	89.9
11	Post-traumatic	Femur	55	87.0	89.0	95.0	95.0	89.0	89.0	86.5	83.5
13	Congenital	Femur	50	11.5	118.5	85.0	87.5	85.5	88.0	81.5	85.5

14	Post-traumatic	Femur	80	96.0	96.0	85.0	86.5	85.0	87.5	94.5	92.0
15	Post-traumatic	Femur	40	74.0	83.5	81.5	81.0	78.5	80.5	90.5	88.5
16	Post-traumatic	Femur	70	110.5	121.0	83.0	82.5	95.5	83.0	80.0	81.5
17	Post-traumatic	Femur	50	115.0	110.0	106.0	94.0	106.0	95.0	90.0	85.0
18	Congenital	Tibia	65	83.7	105.9	81.3	83.3	84.4	84.7	89.9	95.1
19	Congenital	Femur	65	83.5	96.5	82.5	83.5	82.0	83.0	86.5	88.5
19	Post-traumatic	Femur	70	91.5	108.0	78.5	82.0	84.5	84.0	91.5	90.0
21	Congenital	Femur	30	84.5	94.0	82.0	84.0	82.0	85.0	89.5	93.5
22	Post-traumatic	Femur	35	93.5	97.0	88.5	87.5	89.0	87.0	91.0	89.0
23	Post-traumatic	Femur	40	84.5	84.5	93.7	94.4	100.4	89.2	82.9	85.5
24	Post-traumatic	Femur	65	87.9	91.7	90.7	88.7	89.9	89.6	95.7	94.4
25	Post-traumatic	Femur	25	88.7	90.9	100.7	89.2	84.9	85.0	91.3	92.8
26	Post-traumatic	Femur	55	90.8	95.3	90.3	94.0	92.4	90.2	85.9	85.1
27	Post-traumatic	Femur	50	90.4	96.3	88.0	89.8	86.6	87.4	84.5	85.3
28	Congenital	Femur	80	83.2	86.0	88.8	87.7	85.7	86.9	68.8	79.8
29	Congenital	Tibia	60	77.3	84.2	82.8	83.3	88.3	81.6	71.9	84.5
30	Congenital	Tibia	50	83.1	90.2	86.5	87.3	88.2	86.5	84.8	87.5
31	Congenital	Femur	35	91.0	94.2	99.7	100.8	93.8	93.4	80.8	82.5
32	Post-traumatic	Femur	70	86.1	85.8	90.4	91.9	87.1	87.0	82.6	83.1
33	Post-traumatic	Femur	55	94.3	95.2	87.1	93.3	91.6	90.1	89.4	87.5

34	Congenital	Femur	70	121.1	130.7	73.9	81.7	93.1	91.4	90.9	84.4
35	Post-traumatic	Femur	50	86.8	89.0	79.7	82.0	84.0	83.6	83.0	90.1
36	Post-traumatic	Femur	26	90.0	90.0	90.0	90.0	90.0	90.0	90.0	90.0
37	Congenital	Femur	50	124.3	127.9	92.7	90.0	90.8	96.3	84.8	88.8

Abbreviations: LLD, limb length discrepancy; mLPFA, mechanical lateral proximal femoral angle; mLDFA, mechanical lateral distal femoral angle; mMPTA, mechanical medial proximal tibial angle; mLDTA, mechanical lateral distal tibial angle.
